# Apalutamide‐associated skin rash in patients with prostate cancer: Histological evaluation by skin biopsy

**DOI:** 10.1002/iju5.12331

**Published:** 2021-06-27

**Authors:** Yoichiro Tohi, Koki Kataoka, Yumi Miyai, Yo Kaku, Teruki Dainichi, Reiji Haba, Hiroyuki Tsunemori, Mikio Sugimoto

**Affiliations:** ^1^ Department of Urology Faculty of Medicine Kagawa University Kagawa Japan; ^2^ Department of Dermatology Faculty of Medicine Kagawa University Kagawa Japan; ^3^ Department of Diagnostic Pathology Faculty of Medicine Kagawa University Kagawa Japan

**Keywords:** adverse event, apalutamide, eczematous drug eruption, prostate cancer, skin rash

## Abstract

**Introduction:**

Apalutamide‐associated skin rash is a more common adverse event in the Japanese population than in the global population. However, its mechanism remains elusive, and limited histopathological information hampers further understanding.

**Case presentation:**

Case 1: a 71‐year‐old man with metastatic castration‐sensitive prostate cancer developed a skin rash after 70 days of apalutamide treatment. Case 2: a 71‐year‐old man with non‐metastatic castration‐resistant prostate cancer developed a skin rash after 71 days of apalutamide treatment. In both cases, the skin rash presented as a slightly exudative erythema. The histology showed spongiosis of the epidermis and perivascular and interstitial infiltration of lymphocytes and eosinophils in the upper dermis without necrotic keratinocytes.

**Conclusion:**

Apalutamide‐induced skin rashes can involve an eczematous reaction clinically and histologically.

**Abbreviations & Acronyms**
CTCAE: National Cancer Institute Common Terminology Criteria for Adverse Events
DRESS: drug rash with eosinophilia and systemic symptoms
HE: hematoxylin‐eosin
mCSPC: metastatic castration‐sensitive prostate cancer
nmCRPC: non‐metastatic castration‐resistant prostate cancer
SAEs: severe adverse events
SJS: Stevens‐Johnson syndrome
TEN: toxic epidermal necrolysis

**Keynote message**

Our two cases suggest that apalutamide‐associated skin rash can be attributed to an eczematous‐type drug eruption. Our cases provide insight into histopathological information on apalutamide‐associated skin rash.

## Introduction

Apalutamide is a next‐generation antiandrogen for the treatment of nmCRPC and mCSPC.^1^, ^2^ Apalutamide treatment demonstrated the prolonged metastatic‐free survival and overall survival in phase III studies, and is one of the first‐line drugs for nmCRPC and mCSPC.^1^, ^2^ A skin rash is a more common adverse event in Japanese patients (51.5%) than in the global population (SPARTAN study, 23.8%; TITAN study, 27.1%).^1^, ^2^, ^3^ In most cases, the rash is dose‐dependent, and requires dose reduction or discontinuation during treatment in the affected cases.^4^ However, the mechanism of this common adverse event remains elusive, and limited histopathological information hampers further understanding. We present two representative cases of apalutamide‐associated skin rash that was evaluated histologically.

## Case presentation

### Case 1

**Fig. 1:**
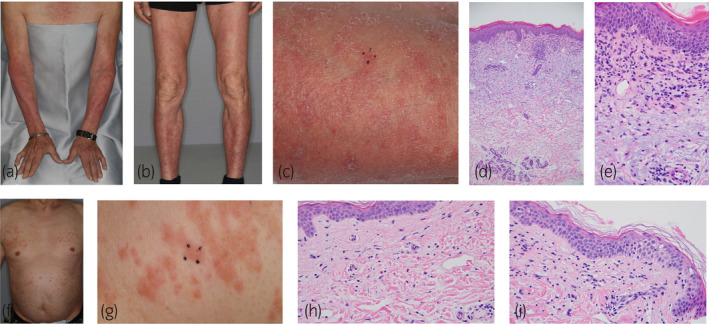
Clinical and histological features: case 1 (a–e) Skin eruption on both forearms (a) and legs (b). The erythema showed a maculopapular pattern with slight infiltration. A biopsy specimen was obtained from the skin surrounded by a black spot on the forearm (c). Histological examination of the biopsy revealed superficial and perivascular infiltration of lymphocytes in the upper dermis (HE staining, ×10) (d). A high‐power view of the specimen revealed eosinophilic infiltration in the upper dermis (e) (HE, ×40). Case 2 (f–i) Skin eruptions on the chest and abdomen (f). A skin biopsy specimen obtained from the chest (g). A high‐power view of the specimen revealed eosinophilic infiltration in the upper dermis (h), spongiosis of the epidermis without necrotic keratinocytes (i) (HE, ×40).

**Fig. 2:**
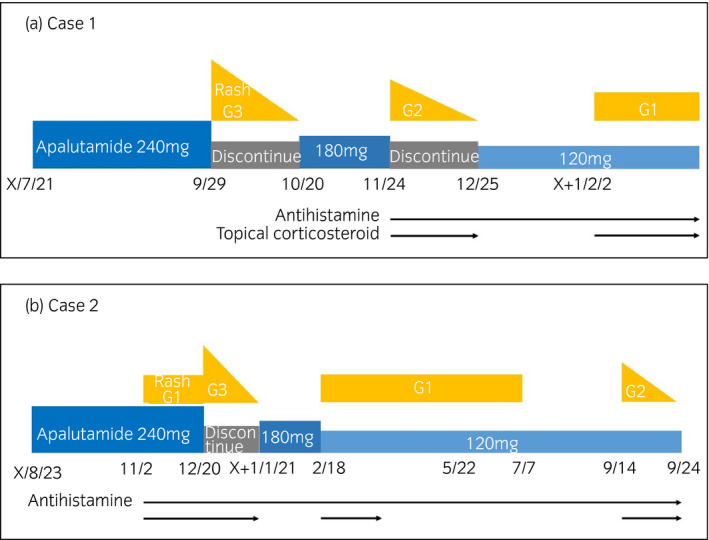
Clinical course: case1 (a), case 2 (b).

A 71‐year‐old man with mCSPC had no allergies, was not taking any medications, and had no comorbidities. Apalutamide was administered orally (240 mg/day) with a gonadotropin‐releasing hormone agonist as first‐line of treatment. Seventy days after initiating apalutamide, the patient developed slightly exudative erythema with pruritus on both forearms, both legs, and chest. The patient did not have high fever, no erosions on mucosa, no sterile pustules, and no Nikolsky’s sign. The erythema was slightly raised, covering 30% of the body’s area, and was classified as grade 2 according to the CTCAE, version 5.0. Laboratory data showed no eosinophilia. No other drugs were changed or added except for apalutamide. The patient scored a 9 on the Naranjo scale; therefore, a causal relationship between the skin rash and apalutamide was strongly suspected.^5^ Apalutamide was discontinued for 3 weeks and restarted at 180 mg/day. Thirty‐five days after re‐administration, the patient developed grade 2 erythema (Fig. 1a–c). A skin biopsy of the forearm revealed spongiosis of the epidermis without necrotic keratinocytes, as well as perivascular and interstitial infiltration of lymphocytes and a few eosinophils in the upper dermis. Vacuolar changes in the dermo‐epidermal interface were not prominent. (Fig. 1d,e). Histological evaluation eliminated SAEs. Apalutamide was discontinued once again, and topical corticosteroids and oral antihistamines were administered to reduce the eruption. Three weeks after the second discontinuation, apalutamide was restarted at 120 mg/day in combination with an oral antihistamine. Six weeks after the third administration, the patient developed grade 1 erythema. The skin eruption was controlled to within tolerable levels for 8 weeks without the need for apalutamide discontinuation (Fig. 2a; Table 1).

**Table 1:** Summary of two cases

Case	Age	Medication history	Disease status	Apalutamide start dose	Time‐to‐first incidence of skin rash	Body part of skin rash	Skin rash management	Time‐to‐re‐administration of apalutamide	Pathological feature of skin rash	Recurrence of skin rash	Apalutamide continuation	Current status
1	71	None	mCSPC	240 mg	70 days	Forearms Legs Chest	Aplutamide discontinue	21 days	Spongiosis of the epidermis without necrotic keratinocytes, as well as perivascular and interstitial infiltration of lymphocytes and a small amount of eosinophils in the upper dermis	Yes	9 months	Aplutamide 120 mg continue
2	71	Azilsartan Eliquis Takelda	nmCRPC	240 mg	71 days	Body Extremities	Aplutamide discontinue	32 days	Spongiosis of the epidermis without necrotic keratinocytes, as well as perivascular and interstitial infiltration of lymphocytes and a small amount of eosinophils in the upper dermis	Yes	14 months	Switch to darolutamide as requested by the patient

### Case 2

A 71‐year‐old man with nmCRPC had no allergies, was taking three medications, and had comorbidities such as cerebral infarction and atrial fibrillation developed a skin rash 71 days after apalutamide treatment. The patient did not have high fever. The skin rash presented as a slightly exudative erythema on the extremities and the body relatively symmetrically without erosions on mucosa and sterile pustules, and were classified as grade 3 according to CTCAE (Fig. 1f,g). There was no Nikolsky’s sign. No other drugs were changed or added, except for apalutamide. A skin biopsy of the chest revealed spongiosis of the epidermis without necrotic keratinocytes, as well as perivascular and interstitial infiltration of lymphocytes and a few eosinophils in the upper dermis. Vacuolar changes in the dermo‐epidermal interface were not prominent. (Fig. 1h,i). A histological evaluation eliminated SAEs, which support continuation of apalutamide. Thereafter, the skin rash improved with discontinuation of apalutamide; however, 49 days after re‐administration with the two dose reductions, the patient developed grade 1 erythema. The skin eruption was controlled to within tolerable levels for 8 months without apalutamide discontinuation in combination with an oral antihistamine. The medication was switched to darolutamide as requested by the patient due to apalutamide‐associated skin rash (Fig. 2b; Table 1).

## Discussion

In our two cases, the clinical features of skin rash induced by apalutamide presented slightly exudative erythema, and the histological features revealed spongiosis without necrotic keratinocytes. These findings were in line with the histological features of a previous report on a skin rash biopsy.^6^ However, in case 2, maculopapular exanthema cannot be ruled out, because erythematous maculopapulous eruption was distributed symmetrically on the body.^7^ Severe adverse cutaneous drug reactions, such as SJS, TEN, and DRESS, were excluded from the differential diagnosis despite a previous fatal case.^8^ Other common adverse cutaneous drug reactions, such as acute generalized exanthematous pustulosis, and fixed drug eruption, were also excluded clinically and histologically.^7^, ^9^ Therefore, previous^6^ studies and our cases suggest that atypical eczematous rash may be a unique adverse reaction to apalutamide whereas further studies are required to categorize the phenotypes of the drug eruptions.

The mechanism of apalutamide‐associated skin rash is poorly understood. Of note, the skin rash has a statistically significant association with a higher dose of apalutamide, or its active metabolite exposure.^4^ In addition, the frequency of the skin rash is much higher during treatment with apalutamide than with other antiandrogens.^10^ These findings prompted us to consider that the apalutamide‐associated skin rash might not be attributed to allergic reactions, but a structure‐specific, off‐target pharmacological reaction.

There is no difference in metastasis‐free survival between the initial dose of 240 mg of apalutamide and the adjusted dose.^4^ These results suggest that dose reduction or discontinuation of apalutamide does not have a negative effect on its efficacy. In a phase III clinical study of apalutamide, body weight was lower in the Japanese population (median 61.9 kg) compared to the global population (median 85 kg).^1^, ^11^ The initial dose of 240 mg of apalutamide may be too high for Japanese patients. Therefore, one possible approach is to start with an adjusted dose to avoid skin rash; however, there is currently no evidence for this approach and further study is needed.

In conclusion, our cases support the idea that eczematous eruption is one of the representative types of apalutamide‐induced skin rash. Clinical and histological features of our cases provide useful information for the management of this common adverse events and enhance the mechanistic understanding in future studies.

## Conflict of interest

MS has received honoraria from Janssen Pharmaceutical K.K. Other authors declare no conflict of interest.

## Approval of the research protocol by an institutional reviewer board

For this type of study, formal consent is not required. This article does not contain any studies with animals performed by any of the authors.

## Informed consent

Informed consent was obtained from the patient included in this study.

## Registry and the registration no. of the study/trial

Not applicable.
